# Molecular Mechanisms of Exercise-induced Hippocampal Neurogenesis and Antidepressant Effects

**DOI:** 10.31662/jmaj.2023-0010

**Published:** 2023-04-07

**Authors:** Makoto Kondo

**Affiliations:** 1 Department of Anatomy and Neuroscience, Graduate School of Medicine, Osaka Metropolitan University, Osaka, Japan

**Keywords:** exercise, hippocampal neurogenesis, antidepressant effects, serotonin, 5-HT3 receptor

## Abstract

It is estimated that approximately 280 million people worldwide suffer from depression. Depression is a common disease to us all, and the socioeconomic loss caused by depression is very large. However, there is currently a problem that many depressed patients do not respond to existing antidepressants, including selective serotonin reuptake inhibitors (SSRIs). Therefore, novel and effective therapeutic agents are highly desirable. It has been reported that exercise has preventive effects on depression (antidepressant effects) and that serotonin, whose release increases in the brain with exercise, is involved in exercise-induced antidepressant effects. We focused on the action of serotonin and investigated its role in the antidepressant effect of exercise using gene knockout mice, and then, we found that serotonin type 3 (5-HT3) receptors play an essential role in the antidepressant effect of exercise. We then further investigated the antidepressant effects mediated by 5-HT3 receptors. Our detailed analyses revealed that neurons expressing 5-HT3 receptors are abundant in the subgranular zone of the hippocampal dentate gyrus and produce insulin-like growth factor-1 (IGF-1). In addition, we newly found that the stimulation of 5-HT3 receptors by agonists promotes IGF-1 release in the hippocampus and increases hippocampal neurogenesis via the IGF-1 signaling pathway, resulting in antidepressant effects. Furthermore, we further showed that a 5-HT3 receptor agonist increases hippocampal neurogenesis and exhibits antidepressant effects in mice with depressive-like behavior. A comparison with the effects of existing antidepressant SSRIs revealed that the 5-HT3 receptor-mediated antidepressant action is a new therapeutic mechanism that differs from existing drugs. Our findings suggest a novel 5-HT3 receptor-IGF-1 mechanism, which could lead to the development of new antidepressant drugs for depression based on the molecular mechanism of exercise-induced antidepressant effects and could bring significant benefits to many depressed patients who do not respond to existing drugs such as SSRIs.

## Introduction

According to a World Health Organization report, there are approximately 280 million depressed people worldwide ^(1)^. Depression is a common mental illness to us all, and the socioeconomic loss caused by depression is very large. The National Health Promotion Movement in the 21st Century (Health Japan 21) launched by the Japanese government emphasizes the importance of the prevention and early treatment of depression from the perspective of mental health. Currently, antidepressants, including selective serotonin reuptake inhibitors (SSRIs), are used for the pharmacological treatment of depression. However, a significant proportion of depressed patients do not achieve remission, and the therapeutic effect is not sufficient ^(2)^. The large number of patients with treatment-resistant depression who do not respond to existing antidepressants has become a major social problem, and there is an urgent medical need to develop novel and effective therapeutic agents for depression ^(3)^.

## Exercise-induced Antidepressant Effects

It is well known that exercise is beneficial for the prevention and improvement of cardiovascular diseases, diabetes, osteoporosis, and so on. Recent studies have revealed that exercise also has beneficial effects on the brain ^(4), (5)^. The effects of exercise on the brain have been reported at the experimental animal and human levels, including the promotion of neurogenesis in the hippocampus ^(6), (7)^, prevention and improvement of depression (antidepressant effects) ^(8), (9)^, and enhancement of learning ability ^(10), (11)^.

Serotonin (5-hydroxytryptamine, 5-HT) is a neurotransmitter that works in the central nervous system to regulate various physiological functions, such as thermoregulation, feeding behavior, sleep-wakefulness, emotion, and memory, and its involvement in depression and hippocampal neurogenesis has been reported ^(12), (13)^. Recently, it was reported that exercise increases the release of serotonin in the hippocampus and that serotonin plays an important role in the increase in hippocampal neurogenesis and antidepressant effects induced by exercise ^(14)^. However, the detailed mechanism of how exercise-induced serotonin increase in the hippocampus promotes hippocampal neurogenesis and produces antidepressant effects has not been clarified ^(15)^.

The serotonin receptor consists of seven subfamilies (5-HT1 to 5-HT7 receptors) ^(16)^. Most of them are G protein-coupled receptors, but the 5-HT3 receptor is the only ionotropic receptor ^(17)^. The 5-HT3 receptor is expressed in the limbic regions of the brain, including the hippocampus, amygdala, and prefrontal cortex ^(18), (19)^, and has been reported to be involved in emotion and memory ^(20), (21), (22)^, but its possible role in hippocampal neurogenesis and depression has not been clarified. Therefore, we sought to investigate the possible relationship of 5-HT3 receptors with exercise-induced increase in hippocampal neurogenesis and antidepressant effects using 5-HT3 receptor knockout (*Htr3a^−/−^*) mice.

## Mechanisms of Exercise-induced Antidepressant Effects

### 1. Exercise-induced increase in hippocampal neurogenesis and 5-HT3 receptors

The hippocampal dentate gyrus is the site where neurogenesis occurs in an adult brain. Many serotonergic neurons project to the hippocampal dentate gyrus ^(23)^, and serotonin is known to promote hippocampal neurogenesis ^(24)^. It has also been reported that hippocampal neurogenesis is important for the antidepressant effects caused by antidepressants and exercise ^(25), (26)^. We first examined the relationship between exercise-induced increase in hippocampal neurogenesis and 5-HT3 receptors.

**Figure 1.**
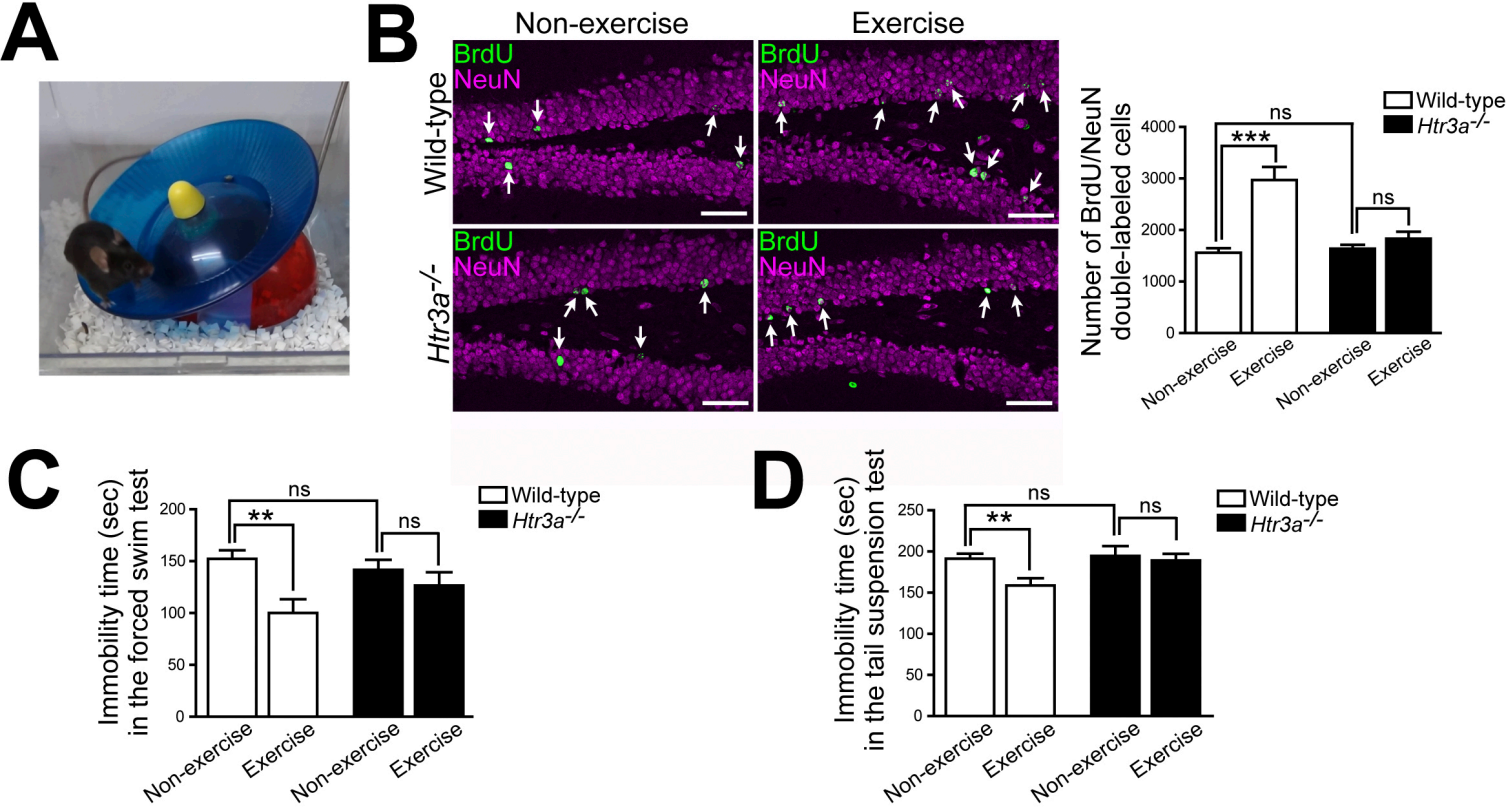
5-HT3 receptors are essential for exercise-induced increases in hippocampal neurogenesis and antidepressant effects. (A) Exercise condition. (B) Exercise for 3 weeks increased BrdU-labeled mature granule cells (BrdU/NeuN double-labeled cells) in the hippocampal dentate gyrus in wild-type mice but not in *Htr3a^−/−^* mice. Scale bars, 50 μm. (C, D) Three weeks of exercise decreased immobility time, indicating antidepressant effects. By contrast, *Htr3a^−/−^* mice showed no decrease in immobility time after exercise for 3 weeks (C: forced swim test; D: tail suspension test). **p < 0.01; ***p < 0.001; ns, not significant. Means ± SEM are shown in all histograms. Modified with permission from ref. 27 and 36.

We labeled dividing cells in mice with bromodeoxyuridine (BrdU) and analyzed neurogenesis in the subgranular zone of the hippocampal dentate gyrus by immunohistochemistry. When wild-type mice were housed in the exercise environment equipped with a running wheel (Figure 1A) for 3 weeks, BrdU-labeled mature granule cells (BrdU/NeuN double-labeled cells) in the hippocampal dentate gyrus increased compared to those of mice housed in nonexercise conditions (Figure 1B). By contrast, exercise did not increase BrdU-labeled mature granule cells in *Htr3a^−/−^* mice (Figure 1B). In addition, there were no differences in total locomotor activity (total number of revolutions of running wheel) between wild-type and *Htr3a^−/−^* mice housed in exercise conditions for 3 weeks. These results indicate that 5-HT3 receptors are essential for the exercise-induced increase in hippocampal neurogenesis ^(27)^.

### 2. Antidepressant effects of exercise and 5-HT3 receptors

Next, mice were housed in exercise conditions for 3 weeks and then subjected to behavioral tests to analyze depressive-like behavior. In wild-type mice, immobility time decreased in the forced swimming test (Figure 1C) and the tail suspension test (Figure 1D) after 3 weeks of exercise, indicating the antidepressant effects of exercise. By contrast, *Htr3a^−/−^* mice showed no decrease in immobility time in either test after 3 weeks of exercise (Figure 1C and 1D), indicating that 5-HT3 receptors are essential for the antidepressant effects of exercise ^(27)^.

These results indicate that 5-HT3 receptors play an essential role in the exercise-induced increase in hippocampal neurogenesis and antidepressant effects. In other words, it was suggested that exercise-induced serotonin increase in the hippocampus induces hippocampal neurogenesis and antidepressant effects via 5-HT3 receptors ^(27), (28)^. Therefore, we next focused on the 5-HT3 receptor-mediated antidepressant effects and examined it in more detail.

## 5-HT3 Receptor-mediated Antidepressant Mechanism

### 1. Effects of 5-HT3 receptor agonists on depressive behavior

**Figure 2.**
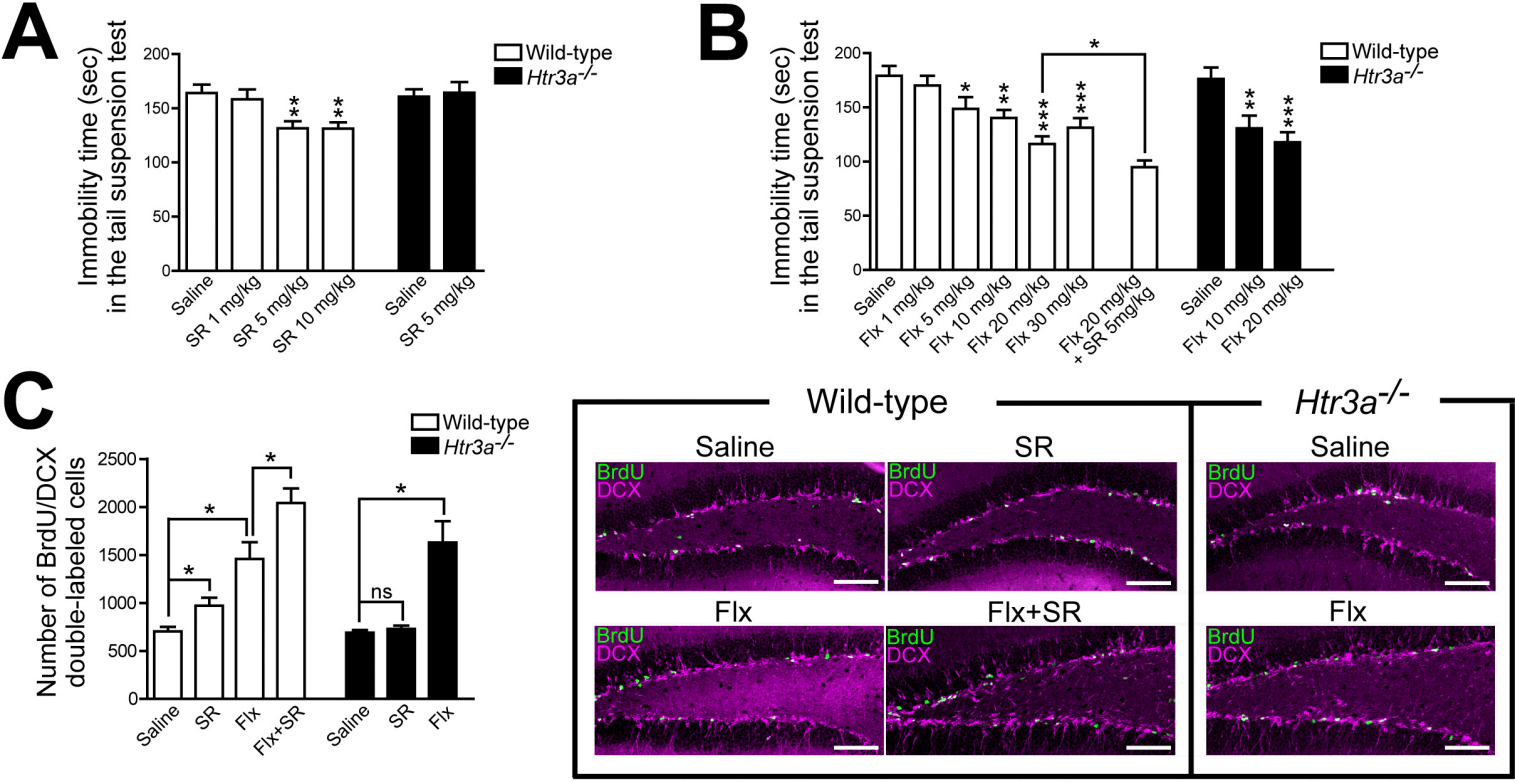
5-HT3 receptor agonists produce increased hippocampal neurogenesis and antidepressant effects through a mechanism different from that of fluoxetine. (A) Treatment of wild-type mice with a 5-HT3 receptor agonist (SR 57227A) produced antidepressant effects. (B) Treatment of wild-type and *Htr3a^−/−^* mice with fluoxetine (a commonly used SSRI) produced a dose-dependent antidepressant effect. Combination treatment of wild-type mice with fluoxetine (20 mg/kg) and SR 57227A (5 mg/kg) produced antidepressant effects that exceeded those produced by fluoxetine (20 mg/kg) alone. (C) Treatment of wild-type mice with SR 57227A for 3 days increased neural progenitor cells (BrdU/DCX double-labeled cells) in the hippocampal dentate gyrus. Treatment of wild-type and *Htr3a^−/−^* mice with fluoxetine for 3 weeks increased neural progenitor cells. Combined treatment of wild-type mice with fluoxetine and SR 57227A increased more neural progenitor cells than fluoxetine alone. Scale bars, 100 μm. SR, SR 57227A; Flx, fluoxetine; *p < 0.05; **p < 0.01; ***p < 0.001; ns, not significant. Means ± SEM are shown in all histograms. Modified with permission from ref. 29 and 36.

First, we examined the effects of 5-HT3 receptor stimulation with agonists on depressive-like behavior. The administration of a 5-HT3 receptor agonist (SR 57227A) to wild-type mice decreased immobility time in the tail suspension test and produced antidepressant effects (Figure 2A). By contrast, *Htr3a^−/−^* mice showed no decrease in immobility time (Figure 2A).

To further analyze the antidepressant effects of 5-HT3 receptor agonists, we compared their antidepressant effects with those of existing antidepressants, SSRIs. The administration of fluoxetine (a commonly used SSRI) to wild-type mice decreased immobility time in a dose-dependent manner, with a maximal antidepressant effect at 20 mg/kg (Figure 2B). Interestingly, treatment of *Htr3a^−/−^* mice with fluoxetine produced antidepressant effects comparable to those of wild-type mice (Figure 2B). Moreover, the combined administration of fluoxetine (20 mg/kg) and SR 57227A (5 mg/kg) to wild-type mice produced antidepressant effects that exceeded those obtained with fluoxetine (20 mg/kg) alone (Figure 2B). These results suggest that 5-HT3 receptors are not involved in the antidepressant effects produced by fluoxetine and that 5-HT3 receptor agonists produce antidepressant effects by a mechanism different from that of fluoxetine ^(29)^.

### 2. Effects of 5-HT3 receptor agonists on hippocampal neurogenesis

Previous studies have reported that enhanced hippocampal neurogenesis is involved in antidepressant effects ^(25), (26)^. Therefore, we investigated the effects of a 5-HT3 receptor agonist (SR 57227A) on hippocampal neurogenesis in comparison with those of an SSRI (fluoxetine). Neural progenitor cells in the hippocampal dentate gyrus were analyzed by immunohistochemistry using the BrdU labeling of dividing cells. Treatment of wild-type mice with SR 57227A for 3 days increased the number of neural progenitor cells (BrdU/DCX double-labeled cells) in the hippocampal dentate gyrus but not in *Htr3a^−/−^* mice (Figure 2C). By contrast, fluoxetine did not increase dividing cells in wild-type mice after 3 days of treatment but increased progenitor cells after chronic treatment for 3 weeks (Figure 2C). This is consistent with previous studies reporting that SSRIs require several weeks of chronic administration to increase hippocampal neurogenesis ^(30)^. In *Htr3a^−/−^* mice, 3 weeks of fluoxetine treatment increased neural progenitor cells to the same extent as that in wild-type mice (Figure 2C). Furthermore, when wild-type mice were treated with a combination of fluoxetine and SR 57227A, there was an increase in neural progenitor cells to a greater extent than that with fluoxetine alone (Figure 2C). These results suggest that 5-HT3 receptors are not involved in the increase in hippocampal neurogenesis induced by fluoxetine and that 5-HT3 receptor agonists increase hippocampal neurogenesis by a mechanism different from that of fluoxetine ^(29)^.

### 3. Mechanisms of 5-HT3 receptor-mediated increases in hippocampal neurogenesis and antidepressant effects

What then are the mechanisms of 5-HT3 receptor-mediated increases in hippocampal neurogenesis and antidepressant effects that are different from those of fluoxetine?

**Figure 3.**
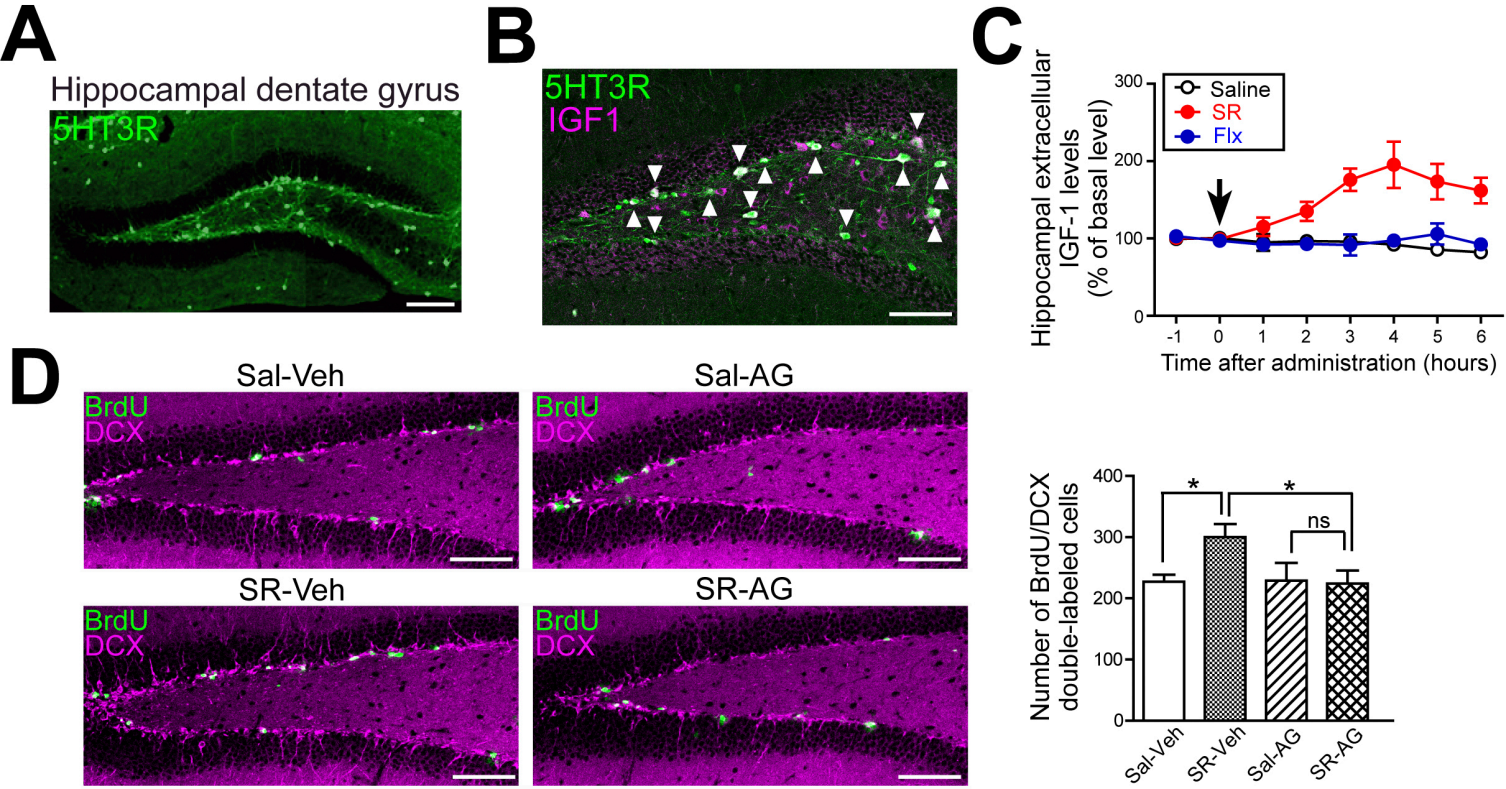
5-HT3 receptor agonists promote IGF-1 release in the hippocampus and increase hippocampal neurogenesis. (A) 5-HT3 receptors are highly expressed in neurons in the subgranular zone of the hippocampal dentate gyrus. (B) 5-HT3 receptors and IGF-1 are expressed in the same neurons in the hippocampal dentate gyrus. Arrowheads indicate neurons expressing both the 5-HT3 receptor and IGF-1. (C) The administration of SR 57227A to wild-type mice increased IGF-1 levels in the extracellular fluid of the hippocampus, but fluoxetine did not affect IGF-1 levels. An arrow indicates the administration time. (D) Treatment of wild-type mice with SR 57227A increased neural progenitor cells (BrdU/DCX double-labeled cells) in the hippocampal dentate gyrus, but this increase was blocked by the intrahippocampal administration of an IGF-1 receptor antagonist (AG 1024). 5-HT3R, 5-HT3 receptor; SR, SR 57227A; Flx, fluoxetine; Sal, saline; Veh, vehicle; AG, AG 1024; *p < 0.05; ns, not significant. Scale bars, 100 μm. Means ± SEM are shown in all histograms. Modified with permission from ref. 29, 31, and 36.

First, we analyzed the expression pattern of the 5-HT3 receptors in the hippocampus in detail using 5-HT3 receptor-EGFP reporter mice and found that 5-HT3 receptors are highly expressed in neurons in the subgranular zone of the hippocampal dentate gyrus (Figure 3A) ^(31)^.

Insulin-like growth factor-1 (IGF-1) is a neurotrophic factor that is expressed in the hippocampus ^(32)^, and it has been reported to promote hippocampal neurogenesis and to have antidepressant effects ^(33), (34)^. We focused on IGF-1 and morphologically investigated the relationship between 5-HT3 receptors and IGF-1 in the hippocampal dentate gyrus. The immunohistochemical analysis of the hippocampus using the reporter mice revealed that 5-HT3 receptors and IGF-1 are expressed in the same neurons (Figure 3B). Furthermore, *in situ* hybridization data revealed that most IGF-1-producing cells in the subgranular zone of the hippocampal dentate gyrus express 5-HT3 receptors ^(29)^.

Next, to further investigate the association between 5-HT3 receptors and IGF-1 in the hippocampal dentate gyrus, we analyzed hippocampal IGF-1 release using *in vivo* microdialysis techniques. The administration of SR 57227A to wild-type mice increased IGF-1 levels in the extracellular fluid of the hippocampus (Figure 3C) but did not alter serum IGF-1 levels. Conversely, fluoxetine did not affect IGF-1 levels in the extracellular fluid of the hippocampus (Figure 3C). In *Htr3a^−/−^* mice, SR 57227A treatment did not alter hippocampal extracellular IGF-1 levels. These results indicate that 5-HT3 receptor agonists promote IGF-1 release in the hippocampus and that this phenomenon does not occur with fluoxetine ^(29)^.

We next examined the relationship between 5-HT3 receptor-mediated IGF-1 release and hippocampal neurogenesis. Treatment of wild-type mice with SR 57227A increased neural progenitor cells (BrdU/DCX double-labeled cells) in the hippocampal dentate gyrus, but this increase was inhibited by the intrahippocampal administration of an IGF-1 receptor antagonist (AG 1024) (Figure 3D). These results suggest that the IGF-1 signaling pathway is important for the 5-HT3 receptor-mediated increase in hippocampal neurogenesis and antidepressant effects ^(29)^.

## Conclusions and Future Perspectives

Our studies revealed that 5-HT3 receptors play an essential role in the exercise-induced increase in hippocampal neurogenesis and antidepressant effects ^(27)^. Furthermore, we found that agonist stimulation of 5-HT3 receptors promotes IGF-1 release in the hippocampus and increases hippocampal neurogenesis via the IGF-1 signaling pathway, resulting in antidepressant effects ^(29)^. This is a new therapeutic mechanism different from existing antidepressant SSRIs. We have also confirmed that the administration of a 5-HT3 receptor agonist to a depression model mouse induced by lipopolysaccharide promotes hippocampal neurogenesis and improves depressive-like behavior ^(29)^. Our findings are expected to lead to the development of novel therapeutic agents for depression based on the molecular mechanism of exercise-induced antidepressant effects ^(35), (36)^. In the future, we would like to build on the results of this study to conduct further research on the novel antidepressant mechanism.

## Article Information

This article is based on the study, which received the Medical Research Encouragement Prize of The Japan Medical Association in 2022.

### Conflicts of Interest

None

### Sources of Funding

This work was supported by JSPS KAKENHI Grant Numbers JP19K11440 and JP22K11498 and by AMED Grant Numbers JP21wm0525026 and JP20lm0203007.
